# Leiomyosarcoma of the abdomen and retroperitoneum; a systematic review

**DOI:** 10.3389/fsurg.2024.1375483

**Published:** 2024-07-17

**Authors:** Mari Nanna Øines, Henry George Smith, Louise Preisler, Luit Penninga

**Affiliations:** ^1^ Department of Surgery and Transplantation, Rigshospitalet, Copenhagen University Hospital, Copenhagen, Denmark; ^2^ Abdominalcenter K, Bispebjerg Hospital, Copenhagen, Denmark; ^3^ Department of Clinical Medicine, University of Copenhagen, Copenhagen, Denmark

**Keywords:** leiomyosarcoma, retroperitoneal tumours, retroperitoneal sarcoma, abdominal sarcoma, abdominal tumours

## Abstract

**Background:**

Intraabdominal and retroperitoneal leiomyosarcomas are rare cancers, which cause significant morbidity and mortality. Symptoms, treatment and follow up differs from other cancers, and proper diagnosis and treatment of intraabdominal and retroperitoneal leiomyosarcomas is of utmost importance. We performed a systematic review to collect and summarize available evidence for diagnosis and treatment for these tumours.

**Methods:**

We performed a systematic literature search of Pubmed from the earliest entry possible, until January 2021. Our search phrase was (((((colon) OR (rectum)) OR (intestine)) OR (abdomen)) OR (retroperitoneum)) AND (leiomyosarcoma). All hits were evaluated by two of the authors.

**Results:**

Our predefined search identified 1983 hits, we selected 218 hits and retrieved full-text copies of these. 144 studies were included in the review.

**Discussion:**

This review summarizes the current knowledge and evidence on non-uterine abdominal and retroperitoneal leiomyosarcomas. The review has revealed a lack of high-quality evidence, and randomized clinical trials. There is a great need for more substantial and high-quality research in the area of leiomyosarcomas of the abdomen and retroperitoneum.

**Systematic Review Registration:**

PROSPERO, identifier, CRD42023480527.

## Introduction

Soft tissue sarcomas are rare tumours that represent a broad and diverse type of cancers that can occur nearly anywhere in the body. These tumours account for less than 1% of all cancers (1). They originate from mesenchymal stem cells, which are present in muscles, fat and connective tissue (1). Soft tissue sarcomas are most frequently located in the extremities, though about 40% are located intraabdominally or retroperitoneally (2). The most common intraabdominal and retroperitoneal soft tissue sarcomas are gastrointestinal stromal tumours (GIST), leiomyosarcomas (LMS) and liposarcomas (LS) (1, 3).

Leiomyosarcoma account for up to 25% of all newly diagnosed soft tissue sarcomas (4, 5). Other types of leiomyosarcoma include those of cutaneous origin, vascular origin, of bone, and in the immunocompromised host. Leiomyosarcomas of vascular origin are also found in the abdomen and retroperitoneum, e.g., leiomyosarcoma of the caval vein. In a Danish prospective cohort study of intraabdominal and retroperitoneal sarcomas, 11% of the tumours were leiomyosarcomas, 39% were GIST, 18% were liposarcomas and 30% had a different histological origin (1).

Intraabdominal and retroperitoneal leiomyosarcomas are rare cancers, which cause significant morbidity and mortality. Symptoms, treatment and follow up differs from other cancers, and proper diagnosis and treatment of intraabdominal and retroperitoneal leiomyosarcomas is of utmost importance. We performed a systematic review to collect and summarize the available evidence for diagnosis and treatment of these tumours.

## Methods

### Study design

This systematic review followed the PRISMA extension guidelines for systematic reviews (PRISMA-P). We prepared a protocol, which was registered in the Prospero Database with registration number: CRD42023480527.

### Participants

Inclusion criteria were: randomized controlled trials (RCTs), reviews, prospective studies, observational studies and case series (*n *≥ 2) reporting on adults treated for histologically confirmed leiomyosarcoma in the abdomen and retroperitoneum. We excluded case reports.

### Outcome measures

We assessed the following outcomes: different aspects of diagnosis and treatment of abdominal and retroperitoneal leiomyosarcoma. This included diagnostic accuracy, treatment modalities and their effect on survival, cancer-related survival, recurrence of disease, adverse effects and harms of treatment, and quality of life.

### Search method for identification of studies

We searched PubMed and Cochrane for relevant studies from the earliest entrance date possible up until January 2021, using the search phrase; (((((colon) OR (rectum)) OR (intestine)) OR (abdomen)) OR (retroperitoneum)) AND (leiomyosarcoma), including mesh terms to obtain titles and abstracts that could be relevant for the review.

### Data extraction

Using Covidence, each hit was systematically reviewed by two of the authors (MØ and LuP) on title and abstract level to exclude irrelevant studies. A second screening process was carried out, where full-text articles were read in order to make a final decision on inclusion of studies. Data was extracted by predefined data-charts: Title, author, year of publication, demographic data, setting, follow-up and results. Inclusion criteria were applied independently by two reviewers, and in case of disagreement, a consensus was reached. Relevant references from included studies were also included. References were managed using Mendeley®.

## Results

**Figure 1:**
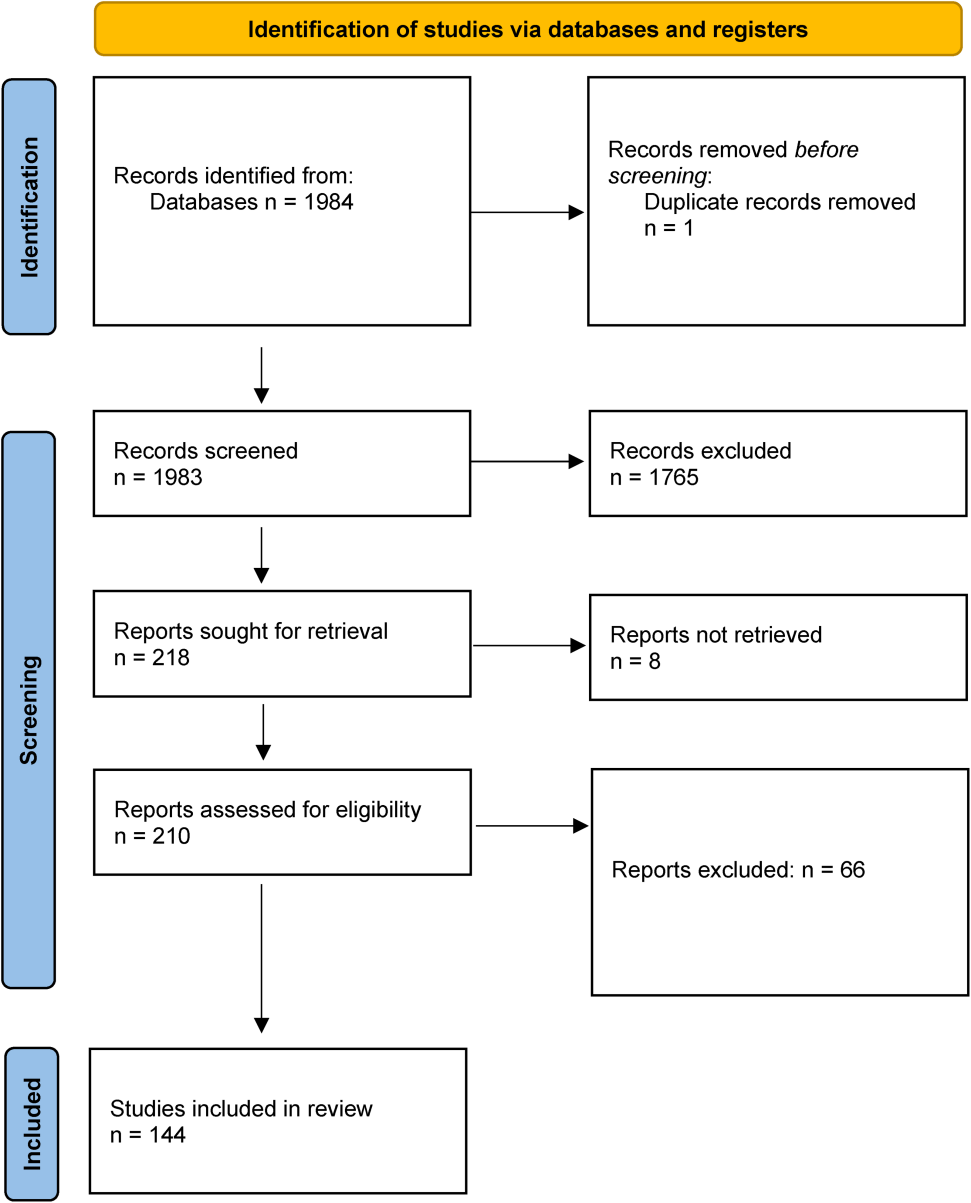
PRISMA diagram.

Our predefined search identified a total of 1,983 publications, of which 218 were selected and retrieved in full-text (Figure 1). 144 were ultimately included in the review. The studies are summarized in Supplementary Material Table S1 (see Supplementary Material). There are 108 publications regarding leiomyosarcoma of the abdomen, of which 75 were abdominal tumours only, while the rest included multiple locations. There are 64 publications regarding retroperitoneal leiomyosarcomas, of which 22 were retroperitoneal tumours only. 55 studies reported on leiomyosarcomas only, while the rest included multiple histologies, both malignant and benign. The primary reason for study exclusion were case reports, leiomyosarcoma of other locations than the abdomen and retroperitoneum (uterine e.g.,), non-human studies and *in vitro* trials.

### Study characteristics

We found 1 randomized controlled trial investigating neoadjuvant radiotherapy in patients with resectable leiomyosarcoma/soft tissue sarcoma (6), and 1 prospective study reporting the incidence of sarcoma in a population (7). All other included studies were retrospective cohort studies, review articles, case series and guidelines.

### Analysis and statistics

We performed a systematic literature review of publications concerning leiomyosarcoma of the abdomen and retroperitoneum to write this systematic review. The literature was evaluated and reported in a systematic fashion in our review. We intended to pool data from included studies if possible, though the available studies were too diverse to pool results and perform a meta-analysis, because they mostly describe leiomyosarcomas of multiple anatomical locations or multiple types of sarcomas in the same anatomical location (i.e., abdomen or retroperitoneum). Furthermore, interventions and outcomes differ between studies, which also make in depth comparison difficult. This is why there is no metaanalysis, further statistical analysis or description of additional statistics.

### Epidemiology

The most common sites of leiomyosarcoma are the abdomen or retroperitoneum, uterus and extremities (5). Approximately 50% of all leiomyosarcomas are located in either the abdomen or retroperitoneum (2).

In a French register study of sarcoma incidence from 2000 to 2013, the male to female ratio of leiomyosarcoma was 0,6, while the overall male to female ratio of sarcomas in intestinal organs was 1,0 (8). In a Danish study from 2011, 53% of abdominal and retroperitoneal sarcomas were found in men, and 47% in women (1). According to the latest annual report of the Danish Sarcoma Database, 50,8% of all sarcomas were found in men, and 49,2 in women (9).

Sarcomas most commonly occur after 40 years of age. According to the French register study, sarcoma incidence was highest in the age-group 40–64 years (35,7%), followed by those aged 75 and above (27,4%) and 65–74 (19,2%) (8). In Denmark in 2019, the sarcoma incidence was 21,4% among both patients of 61–70 years and 71–80 years, while those aged 51–60 years had an incidence of 14%, and 41–50 years and 80 + an incidence of 10,7% (9).

### Abdominal leiomyosarcoma

The predominant intraabdominal locations of leiomyosarcoma are the small and large intestine, but the tumour can also be oesophageal or gastric (10). In addition, a whole range of rare locations have been described in published cases, including the gallbladder, liver, Meckel's diverticulum, porta hepatis, pancreas, spleen, appendix, and various blood vessels like the iliac vein.

Only 0,1% of colorectal malignancies are sarcomas (11), and of these, some 90% are leiomyosarcomas (12). There is a connection between previous radiation therapy and the development of anorectal leiomyosarcoma, and a review of published cases showed an incidence of 11,7% of radiation-induced leiomyosarcoma in this subpopulation (13).

### Retroperitoneal leiomyosarcoma

Leiomyosarcoma is the second most common type of retroperitoneal sarcomas, with an incidence of approximately 20%, while liposarcoma, the most common type of retroperitoneal sarcoma has an incidence of 64% (14, 15).

As mentioned above, leiomyosarcoma can occur in various blood vessels, and is common in retroperitoneal located blood vessels like the caval vein, and occasionally in the iliac vein. Leiomyosarcomas of the caval vein are classified in three groups according to Mingoli et al. Segment 1 caval vein LMS are located from the aortic bifurcation to the infrarenal veins. Segment 2 LMS are located from the interrenal or suprarenal veins to, but not involving the main hepatic veins, while Segment 3 LMS involve the main hepatic veins and extends to the right atrium or extends into the heart (16). Approximately 25%–37% of intravascular cases involve segment 1. Segment 2 is the most common site of disease, accounting for 43%–69% of intravascular cases. Segment 3 is the least commonly affected segment, representing 6%–20% of intravascular cases (17, 18).

### Clinical presentation

Symptoms of leiomyosarcoma of the abdomen and retroperitoneum vary greatly depending on tumour site. There might be diffuse symptoms or no symptoms at all. Depending on tumour location, there might be haemorrhage, pressure symptoms, pain or ascites (1). According to Clark et al., the most common finding at diagnosis is a painless, gradually enlarging mass (19). Some patients primarily present with weight loss and abdominal pain, other with intestinal obstruction and dysphagia. While unspecific, anaemia is also a possible symptom (20).

### Diagnosis

The definitive diagnosis of leiomyosarcoma, and other sarcomas, should involve a broad multidisciplinary team of pathologists, radiologists, surgeons, radiation therapists and medical oncologists, preferably at specialist centres (3, 14).

The National Comprehensive Cancer Network (NCCN) guidelines for intraabdominal and retroperitoneal soft tissue sarcoma, recommends CT of the chest, abdomen and pelvis with intravenous contrast for diagnosis, occasionally supplemented by MRI of lesions in the pelvis or abdomen. PET/CT can be considered in order to detect distant metastases, or to help determine the site of biopsy (21).

According to the European Society for Medical Oncology-European Reference Network for rare adult solid cancers (ESMO-EURACAN) report on soft tissue and visceral sarcomas from 2018, all retroperitoneal tumours should be biopsied. The risk of needle track seeding is minimal, if the biopsy is thoroughly planned, and not performed transperitoneally (3). Similarly, a consensus statement on retroperitoneal sarcoma from 2021 strongly recommends image-guided core needle biopsy to secure the reliability of the diagnosis, and allow for histologic and molecular subtyping and grading. The risk of needle tract seeding during this procedure is not zero, but very low, and the benefits of proper preoperative diagnostics are considered to greatly outweigh the risks (14).

Recommendations from the NCCN argue that image guided core needle biopsy should be performed if preoperative treatment is planned, or if non-sarcoma malignancies are suspected. If the tumour is a well differentiated liposarcoma, biopsy is unnecessary. The rationale for biopsy is to determine whether the tumour is malignant or benign, provide a specific diagnosis if possible, and determine tumour grade where appropriate. For some non-sarcoma malignancies, like lymphoma or germ cell tumours, first choice of treatment is not surgical, and a preoperative biopsy can prevent unnecessary surgical procedures. Furthermore, biopsies should be examined by pathologists with special expertise in sarcomas (21).

### Histopathology

Leiomyosarcoma is a malignant mesenchymal tumour of smooth muscle origin. Histologically, it is characterized by the presence of spindle cells with abundant eosinophilic cytoplasma and hyperchromatic nuclei. There can be necrotic areas in the tumour, and areas of pleomorphism (22). The criteria for malignancy are mitotic activity of more than 2 MF/50 HPF (mitotic figures/high power field) and nuclear atypia (22).

Immunohistochemistry is necessary to obtain an accurate diagnosis of leiomyosarcoma. Leiomyosarcoma can be differentiated from other soft tissue sarcomas by the presence of smooth muscle cell actin and desmin on immunohistochemistry. To differentiate leiomyosarcoma from myofibroblastic sarcoma, heavy-caldesmon and smooth muscle myosin can be useful markers (23).

According to the NCCN guidelines on soft tissue sarcoma, there is no ancillary technique to support the morphological diagnosis of leiomyosarcoma (21).

### Staging

**Figure 2:**
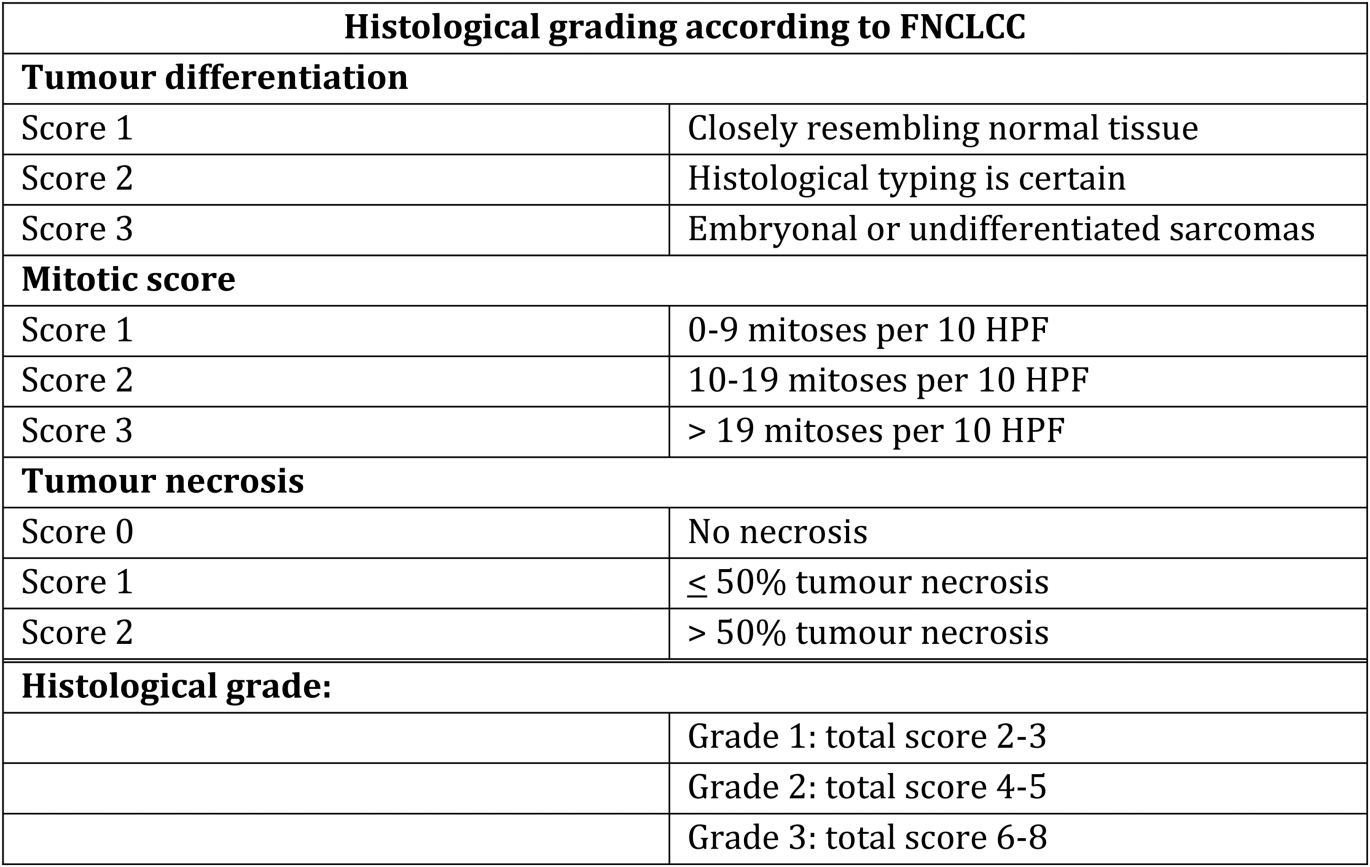
Histological grading.

Leiomyosarcoma can be more or less aggressive, and are classified as malignancy grade 1–3 based on differentiation (1–3), mitoses (1–3) and necrosis (0–2) according to the French Federation of Cancer Centres Sarcoma Group (FNCLCC)-system (24–26). See Figure 2.

A combination of TNM classification and malignancy grade results in a categorization of retroperitoneal tumours in stage 1–4 (3).

Furthermore, tumour size, site, resectability and the presence of metastases are of relevance for proper staging (3). Pathological diagnosis is categorized according to the 2020 WHO classification of Soft Tissue and Bone Tumours (27).

### Genetic subtypes

Gene expression patterns play a role, and may affect tumour characteristics, how sensitive the tumour is for chemotherapy and also affects prognosis (28, 29). Whole-Exome and RNA sequencing of leiomyosarcomas has been performed, and three mRNA expression subtypes have been identified. These subtypes may or may not vary with anatomical location (30–32). Genetic subtype 1 is primarily found in the extremities and gynaecological tumours. Subtype 2 is primarily found in the abdomen, and to a lesser degree in the extremities. While subtype 3 primarily is found in gynaecological leiomyosarcomas, to a lesser degree in the abdomen, but not in the extremities (31, 32).

The distribution of these three genetic subtypes may be explained by the following: Subtype 1 &2 comprises extremity and abdominal leiomyosarcoma, which resembles vascular smooth muscle; Subtype 2 comprises abdominal leiomyosarcoma, which resembles digestive smooth muscle. Subtype 3 comprises gynaecological leiomyosarcoma, which resemble uterine smooth muscle (30).

Genetic studies have also showed a near-universal inactivation of TP53 and RB1 genes, while a homologous recombination (HR)-deficiency signature (SBS3) was present in 98% of all specimens (33).

Another genomic finding is that alteration in muscle related genes differs in the three leiomyosarcoma subtypes. Myocardin (MYOCD) amplifications occur frequently in subtypes 2 and 3, while dystrophin (DMD) gene deletions occur predominantly in subtype 1, and to a lesser degree in subtype 3.

In addition, a high immune infiltration expressed as enrichment of Macrophage M2 is associated with LMS subtype 1, and subtype 1 has also been called inflammatory LMS. In a gene-expression study, Hemming et al. called subtype 1 inflammatory LMS, with a high ARL4C gene expression, and detected a worse disease-specific survival (34). Subtype 2 was called conventional LMS, was muscle-associated with a high Insulin-like growth factor 1 receptor (IGF1R) expression, and subtype 3 was called uterogenic LMS with an uterine-like gene expression profile, and high prolactin expression. Worse survival was associated with subtype 1 compared to subtype 3 for gynaecological cancers, and subtype 2 appears to have the best survival of the three subtypes. It appears that LMS subtypes may play a more important role than LMS location to predict prognosis and survival. This also raises the question whether further trials should be designed based on molecular LMS subtype, and not on LMS location. An argument for this is that DNA-damage response inhibition (DDRi) has been demonstrated to be effective across different locations. Knowledge on the three genetic subtypes also indicates that immunotherapy possibly is most effective in the inflammatory LMS subtype 1.

### Treatment

Treatment options are complex, and a treatment plan should be discussed at a multidisciplinary team conference (3, 21). A recent consensus statement by the Transatlantic Australasian Retroperitoneal Sarcoma Working Group provides evidence of increased survival, reduced postoperative morbidity and mortality, significantly higher adherence to guidelines, and reduced risk of relapse and sarcoma-related death when patients are treated for retroperitoneal sarcoma at sarcoma reference centres (14). Many of the below treatment principles applies to both abdominal and retroperitoneal sarcomas.

The indisputable first line of treatment for localized leiomyosarcoma, is surgery with liberal excision and negative margins (3, 14, 21). The minimal margin considered acceptable might vary depending on preoperative treatment and presence of anatomical barriers limiting the excision (3). A review of anorectal leiomyosarcomas comprising 51 cases, described both wide local excision and radical resection as treatment options. Local recurrence was more common after wide local excision (30%) compared with radical resection (20%), though the total rate of metastasis was just over 50% regardless of the operative treatment option (13).

Wide excision refers to a dissection plane through unaffected normal tissue within the involved compartment. Radical or compartmental resection refers to *en bloc* excision of the entire involved compartment with no reactive tissue or tumor cells at the margin. For retroperitoneal leiomyosarcomas, there is a tendency towards radical or compartmental resection, and some evidence that retroperitoneal liposarcomas should be treated with radical resection (35).

The aim of a complete resection is to achieve negative margins in the histological sample. The width of these margins are not ultimately defined in the literature, but some suggests a margin of 1 cm, or a layer of intact fascia (36). Excessive lymph node resection does not seem to be necessary, as leiomyosarcoma rarely are metastatic to local lymph nodes (37). If the tumour involves or originate from a blood vessel, the proximal and distal end of the resection should have negative margins. Furthermore, it's recommended to resect tumour thrombosis if present, but the evidence grade of this is unknown.

Resections are categorised as R0-2, where R0 represents margins with no residual microscopic disease, R1 shows residual microscopic disease and R2 ushows macroscopic residual disease. According to the NCCN guidelines on soft tissue sarcoma, resection of a whole anatomical compartment is not usually necessary to obtain oncologically appropriate margins, but evidence is inconclusive. While the NCCN guidelines state that the biopsy site should, if possible, always be included in the resection (21), biopsy sites of retroperitoneal sarcomas are usually left *in situ* (38). This makes it even more important to perform the biopsy with a coaxial technique, and with a retroperitoneal approach rather than intraabdominal.

If the pathologist examining the surgical specimen finds a positive margin after primary surgery of soft tissue sarcoma, re-resections are recommended to achieve negative margins, but only if there is no significant impact on functionality, and if the structures adjacent to the margins are not bone, major vessels or nerves (21).

Similar to abdominal leiomyosarcomas, treatment of retroperitoneal leiomyosarcomas is complete surgical tumour resection with negative margins. Whole anatomical compartment resection is a topic of debate, and more recent management of primary retroperitoneal sarcomas is histology-tailored. For leiomyosarcomas, preservation of adherent organs without direct involvement is preferred, while compartment resection including resection of adherent organs is advised for liposarcomas (35).

However, every surgical procedure entails an individual assessment of extensiveness vs. consequence, and consideration of postoperative morbidity due to damage or resection of retroperitoneal structures. The retroperitoneal space is a confined compartment with multiple large vessels and nerve bundles, limited by bone on multiple sides. This makes radical resection more difficult in some cases of retroperitoneal sarcoma, and marginal surgical resections more frequent. Some structures in the retroperitoneal spaced are more readily sacrificed during surgery, like one kidney, parts of the colon, the adrenal gland and the psoas muscle, while other retroperitoneal structures are more frequently spared due to morbidity if resected, like the bladder, pancreas, duodenum, and major vessels or nerves (14, 38).

Retroperitoneal leiomyosarcomas are usually more well-defined than other retroperitoneal tumours, and closely adjacent organs and structures, provided they are not inseparably adherent or invaded, may be spared if the surgeon can still achieve negative margins (14). When leiomyosarcoma arises from a major vein, special attention should be directed to achieve microscopically negative longitudinal margins of the vein of origin. The use of intra-operative frozen sections to achieve this can be advised (16).

The surgical approach to resect leiomyosarcoma of the caval vein depends on the segment involved. Segment 1 and 2 LMS (below the hepatic veins) can be treated by a midline laparotomy or right subcostal abdominal incision. The retroperitoneum is exposed by mobilizing away non-involved organs like the duodenum, pancreatic head, and the right colon. Proximal and distal control of the inferior caval vein should be achieved including lumbar and renal veins. Finally the involved part of the caval vein should be resected (16). After resection the caval vein can be managed with primary repair, ligation, patch repair, or graft reconstruction. Whether the caval vein can be ligated or should be reconstructed depends on the degree of caval obstruction (presence of thrombus and collateral veins), the degree of cardiac stability when clamping the caval vein, and the complexity of the reconstruction. Ligation of the caval vein is often well tolerated. In the beginning the patient may suffer from lower limb oedema, but often aften a few weeks sufficient collaterals have developed, and symptoms disappear.

Surgical resection of segment 3 LMS of the caval vein is very challenging. Resections are associated with a high mortality risk, and these tumours are considered unresectable by traditional surgical techniques (39). Liver explantation, ex-vivo resection of the retro-and suprahepatic LMS, graft reconstruction of the retrohepatic caval vein, and reimplantation of the liver are amongst the highly specialized surgical options for these tumours. During surgery, venovenous bypass, cardiopulmonary bypass, or portocaval shunting may be required (40). This procedure should be performed at a liver transplant unit, and in the literature only 100 cases have been reported. A ringed polytetrafluoroethylene (PTFE) is the most applied graft for caval reconstruction with good long-term patency (41).

Resection rates of abdominal and retroperitoneal leiomyosarcoma are not readily reported in the published literature. A review of 76 cases of abdominal leiomyosarcoma reported resection rates between 93% - 100% depending on location (20). A Danish register study of abdominal and retroperitoneal soft tissue sarcoma reported a resection rate of 89% for primary sarcomas over a 10-year period. 79% of patients with first recurrence of sarcoma were resectable. Only 11% of the tumours were leiomyosarcomas (1).

A referral centre in Italy published data on patients with inoperable primary retroperitoneal sarcomas, and reasons for not performing surgery. They reported a resection rate of 88,5% over a 4-year period. The primary reason for not performing surgery was a technically non-resectable tumour. The second reason was patient factors such as poor performance status and comorbidities. Approximately 25% of the non-resectable patients had leiomyosarcoma, while 50% had liposarcoma (42). A similar study reported a resection rate of 74% on patients with primary retroperitoneal sarcomas. The reasons for not performing surgery were non-resectability, rapid progression before/under radiotherapy, and poor performance status or comorbidity (43).

There has been an increase in use of adjuvant radiotherapy in some soft tissue sarcomas, including retroperitoneal sarcomas, over the last 5–7 years, while chemotherapy usually has been reserved for stage 4 (metastatic disease) (44, 45). A review of 51 patients with anorectal leiomyosarcomas found that neoadjuvant radiotherapy was associated with a lower risk of local recurrence compared to adjuvant radiotherapy, and also that neoadjuvant radiotherapy facilitates R0 resection of the tumour (13).

In a retrospective review of prognostic factors, 42 patients with intraabdominal or retroperitoneal leiomyosarcoma were included. The patients underwent surgical resection with curative intent, and amongst other prognostic factors, the authors found no impact of adjuvant therapy on survival (46).

In a large retrospective study of more than 7,000 patients with leiomyosarcoma in the National Cancer Database, Gootee et al. found decreased mortality when comparing adjuvant or neoadjuvant radiotherapy in combination with surgery, to surgery alone (4). More than 1,500 patients had leiomyosarcoma of the abdomen, but separate analyses of the effects of chemotherapy on these patients were not performed.

The NCCN guidelines from 2021 on soft tissue sarcoma of the abdomen and retroperitoneum, state that postoperative radiotherapy is not routinely recommended for R0-2 resections. If anything, the surgeon should consider a re-resection if a R0 resection is possible. If surgery leaves a margin close to soft tissue, or a microscopically positive margin, and a R0 resection is not feasible due to anatomical constraints, radiotherapy should be considered. In patients that have received neoadjuvant radiotherapy, a booster dose might be considered postoperatively (21).

In patients with stage IV intraabdominal or retroperitoneal sarcoma, watchful waiting is recommended if the patient is asymptomatic. In symptomatic cases, chemotherapy and/or radiotherapy can be administered, and surgery can be an option to relieve symptoms (21).

There are few randomized trials that explore whether there is an auxiliary effect of concomitant therapy in patients with resectable leiomyosarcoma. Trans-Atlantic Retroperitoneal Sarcoma Working Group (TARPSWG) refers to analyses from the STRASS-1 trial, where 266 resectable patients with retroperitoneal sarcomas from 31 institutions and 13 countries were randomized to either preoperative radiation therapy (RT) followed by surgery, or surgery alone. The RCT showed that there is no evidence that neoadjuvant RT has an impact on local disease control or overall survival, when all histological subgroups are considered. Thus, RT is not routinely recommended for high grade retroperitoneal sarcomas.

Subgroup analysis further revealed that RT was without effect on retroperitoneal leiomyosarcoma, but might play a role in treatment of well differentiated and low-grade dedifferentiated lipomyosarcoma (6, 14).

Neoadjuvant chemotherapy targeted towards specific histological subgroups have shown an increased survival in extremity sarcomas, but these results cannot be extrapolated directly to other soft tissue sarcomas. It is however suggested that neoadjuvant chemotherapy can be facilitated for individual use in patients with chemosensitive histological subtypes, such as retroperitoneal leiomyosarcoma (14). Currently the role of neoadjuvant chemotherapy in patients with retroperitoneal leiomyosarcomas and dedifferentiated liposarcomas is investigated in a multicentre randomized controlled trial in which patients are randomized to neoadjuvant chemotherapy and surgery vs. surgery alone (STRASS-2 trial) (47).

A subgroup analysis of patients receiving perioperative chemotherapy and hyperthermia, showed that this might be beneficial for abdominal sarcomas undergoing R0-1 resections. This treatment is however currently not available in many facilities (14). Postoperative chemotherapy has no beneficial effect after complete en-bloc resection (14).

Postoperative adjuvant chemotherapy can be considered for patients with sarcomas of histological subtypes which have a high tendency for metastatic disease, like leiomyosarcoma. Hypothetically, it makes sense to administer chemo to these patients, since disease relapse is due to hematogenic spread (48).

Previously, Doxorubicin has been the preferred single line treatment for soft tissue sarcoma. Only one trial has demonstrated superiority of treatment with a more extensive regime than single line doxorubicin for metastatic leiomyosarcoma. That trial administered Trabectedine and Doxorubicin in combination, had a median follow up of more than 7 years, but out of 108 patients, only 16 had retroperitoneal sarcoma. Results were reported as progression free survival, which was 12.9 months in the extremity/retroperitoneal group, and overall survival, which was 38,7 months (29).

According to the NCCN guidelines, doxorubicin in combination with ifosfamide is the chemotherapy regimen with the highest response rate in patients with unresectable soft tissue sarcoma (21).

### Prognosis

The 5-year survival of patients in Denmark with primary intraabdominal or retroperitoneal sarcoma is 70,2%. Not surprisingly R0 resections result in a higher 5 year survival of 76,8%, while patients with R1 and R2 resections have a survival rate of 43,5% (1).

Intraabdominal and retroperitoneal leiomyosarcomas have a shorter disease-free survival (DFS) and overall survival (OS), than leiomyosarcomas at other anatomical locations. One study found a 5-year DFS of 39,1% and 35,3% for abdominal and retroperitoneal leiomyosarcomas respectively (46). It also found a 10-year OS of 63,4% for patients with leiomyosarcoma in the abdomen and retroperitoneum, compared to an OS of 79,2% for disease outside the abdomen. Recurrent disease was more often due to metastases in the abdominal/retroperitoneal group (59,5%), than in patients with primary leiomyosarcoma located elsewhere 32,2% (46). The outcome for retroperitoneal leiomyosarcomas may be worse due to large tumour size at diagnosis (median 20 cm), high recurrence rates, and anatomical constraints of retroperitoneal surgery (49).

Other studies have suggested worse outcome for metastatic or recurrent disease with uterine leiomyosarcomas compared to non-uterine leiomyosarcomas, even though uterine leiomyosarcomas were thought to be more sensitive for chemotherapy.

Tumour grade, size, depth and primary site are significant prognostic markers for survival and recurrence. Size and margin status is significant for the rate of local recurrence, while size and grade are relevant for distant recurrence (4, 50).

In a retrospective review of 144 patients with abdominal or retroperitoneal leiomyosarcoma from New York, the 5-year disease free survival of patients was 67%, significantly lower than leiomyosarcomas at other anatomical locations (50). There was a recurrence rate of 51%, which also was higher than for leiomyosarcomas located elsewhere. Distant recurrence was the most common recurrence for leiomyosarcoma at all anatomical sites (53%), but local recurrence was more common amongst patients with intraabdominal or retroperitoneal tumours (30%), than at other anatomical locations (50).

When compared to more common cancers, such as colorectal adenocarcinoma, colorectal leiomyosarcoma has a significantly lower overall 5 year survival rate of 43,8% against 52,3% (11).

Depending on the study, the reported 5 year disease-free survival ranges from 39,1% (46) to 67% for abdominal leiomyosarcoma (50) [56.4% (4)] Given this discrepancy, the reader will appreciate the degree of divergence in published articles on the subject. Reported data is retrospective, sometimes incomplete, and occasionally confounded by inclusion of other sarcomas in the material (predominantly GIST). Furthermore, publications are heterogenous in the sense that some group abdominal and retroperitoneal leiomyosarcomas, while others include uterine and non-visceral sarcomas in their statistics.

In a study of more than 7,000 patients with leiomyosarcoma from the National Cancer Database, age was identified as an independent prognostic factor. The younger the patient was at the time of diagnosis, the better the survival statistics. The authors reported a 3% increase in mortality per additional year of age (4). The patient group was homogenous, and there were no subgroup analysis of the effect of age on abdominal leiomyosarcoma specifically.

### Surveillance

The NCCN guidelines recommend periodical follow up by imaging of the primary site after neoadjuvant therapy, postoperatively and periodically based on the risk of recurrence. Chest imaging by x-ray, CT scan, or PET-CT scan is a necessity due to risk of pulmonary metastases.

In patients without radiographic evidence of disease, imaging of the primary tumour site, chest and other sites at risk of metastases (e.g., the liver) is recommended every 3–6 months the first 2–3 years, every 6 months for the next 2 years, and then annually (21).

27% of patients with intraabdominal or retroperitoneal leiomyosarcoma succumb to disease more than 5 years after they are diagnosed (6% disease-specific mortality after 8 years) (50). This strongly suggests that follow up should be more than 5 years for patients with intraabdominal or retroperitoneal leiomyosarcoma.

Despite complete surgical resection of RPS, the risk of recurrence never plateaus. Consequently, these patients should have lifelong follow-up, which is a burden for patients and healthcare resources. Recurrence might be visible on imaging from months to years prior to any symptoms, and follow up should include CT scans as well as a clinical evaluation. Chest scans may be omitted, particularly in patients with low-grade histology (14).

The median time to recurrence is less than 5 years for high grade RPS, and follow up should probably be performed every 3–6 months the first 5 years, and then every year (14).

### Future perspectives

This systematic review has summarized current knowledge and evidence on non-uterine abdominal and retroperitoneal leiomyosarcomas. The review has revealed a lack of high-quality evidence, and a lack of randomised trials. Little is known, but we are gradually building knowledge through increasing data and subclass definition of soft tissue sarcoma, clinical presentation, histological and genetic sarcoma subtypes, surgical strategies, individualized treatment approaches, adjuvant therapy, follow-up and recurrent disease. There is a great need for more substantial and high-quality research in the area of leiomyosarcomas of the abdomen and retroperitoneum. Consensus statements and publications from global sarcoma associations often lack high quality evidence (14). Abdominal and retroperitoneal leiomyosarcomas are rare tumours, and rare tumours require special actions to acquire evidence. There is a great need for prospective studies with relevant clinical and patient reported outcomes. If possible, these studies should be international multicentre randomised studies. Recent international multicentre RCTs on the effect of neoadjuvant radiotherapy (STRASS-1, completed and published) and neoadjuvant chemotherapy (STRASS-2, currently recruiting) in patients with retroperitoneal sarcomas are excellent examples of how to establish firm evidence. Furthermore, all patients should be registered in international clinical registries.

## Conclusions

- Abdominal and retroperitoneal leiomyosarcomas are difficult to diagnose due to vague symptoms, these tumours are therefore often quite advanced or large when diagnosed.
- Adjuvant therapy for abdominal and retroperitoneal leiomyosarcomas is less effective than with other cancer diseases.
- These tumours have a high risk of distant or local recurrence, also after 5 years of disease-free survival.
- Treatment of sarcoma patients by multidisciplinary teams, and with adherence to guidelines, is important for their survival. Thus, updated knowledge of current best practice is essential for any facility treating sarcoma patients.
- Although based on thorough literature review and expert discussions, most consensus articles, guidelines and reports do not focus specifically on abdominal and retroperitoneal leiomyosarcoma. Thus, some of the above recommendations are more general, and covers a broader group of soft tissue sarcomas, or sarcomas also located at other anatomical sites.
- Classification of LMS in three genetic subtypes is a breakthrough, and should cause future trials to be based on molecular subtype, rather than tumour localisation (abdomen/retroperitoneum, extremities, and gynaecological).

144 studies were eventually included in this systematic review from our search (1, 2, 4, 6–8, 10, 11, 13, 16, 20, 39, 41, 42, 50–54, 55–64, 65–73, 74–83, 84–93, 94–103, 104–113, 114–123, 124–133, 134–143, 144–153, 154–163, 164–173, 174–177).

## Data Availability

The original contributions presented in the study are included in the article/**Supplementary Material**, further inquiries can be directed to the corresponding author.
