# Supplementary material for: Leiomyosarcoma of the abdomen and retroperitoneum; a systematic review
Source: Front Surg. 2024 Jul 17;11:1375483. doi: 10.3389/fsurg.2024.1375483 (PMC11288885; doi:10.3389/fsurg.2024.1375483)
Supplement: Supplementary file 1 [file Table1.docx]

Table 1. Table of studies included in the review “Leiomyosarcoma of the abdomen and retroperitoneum; a systematic review”

| Title | Year | Author | Type of sarcoma | Site of sarcoma | Total number of patients | Number of leiomyosarcomas of the abdomen or retroperitoneum | Median tumour size in cm | Mean/median age | Gender | Prognosis/  Survival rate |
| --- | --- | --- | --- | --- | --- | --- | --- | --- | --- | --- |
| Operative strategies for inferior vena cava repair in oncologic surgery. | 2020 | Ruiz, Colby S et al. | Leiomyosarcoma  Renal cell carcinoma  Testicular tumor  Cholangiocarcinoma  Other tumors | Retroperitoneum  (Inferior vena cava) | 52 | 10 | NA | 54 | 46%  female | 30 day 96%  1 year 75%  2 year 65% |
| A pooled analysis of risk factors of surgically treated leiomyosarcoma of the colon in adults. | 2020 | Wang, Yun et al. | Leiomyosarcoma | Abdomen  (Colon) | 41 | 41 | 9 | 56 | 46%  female | 1 year 82%  3 year 61%  5 year 41% |
| Outcomes of palliative-intent surgery in retroperitoneal sarcoma-Results from the US Sarcoma Collaborative. | 2020 | Thalji, SZ et al. | Leiomyosarcoma | Retroperitoneum  Abdomen | 70 | 13 | 22%  <5  16%  5-10  62% >10 | /62 | 59% female | Median OS 10,7 months |
| Clinical characteristics and surgical outcomes of retroperitoneal tumors: a comprehensive data collection from multiple departments. | 2020 | Sassa, Naoto et al. | Leiomyosarcoma  Liposarcoma  MFH | Retroperitoneum | 422 | 16 | 7,2 | /52 | 49% female | 3 year survival 78,4%  5 year survival 72,8% |
| Preoperative radiotherapy plus surgery versus surgery alone for patients with primary retroperitoneal sarcoma (EORTC-62092: STRASS): a multicentre, open-label, randomised, phase 3 trial | 2020 | Bonvalot, Sylvie et al. | Leiomyosarcoma  Liposarcoma  Other | Retroperitoneum | 247 | 38 | 16 | 61 | 48% female | Median OS 4,5 years with surgery + radiotherapy  5 years with surgery only |
| Primary iliocaval leiomyosarcomas: The path beyond surgery. | 2020 | Ong, CJ et al. | Leiomyosarcoma | Retroperitoneum | 30 | 30 | NA | 61 | 53% female | Median OS 41 months  5 year 32% |
| Retroperitoneal Sarcomas: Prognostic Factors and Outcomes of a Series of Patients Treated at a Single Institution. | 2020 | Patkar, Shraddha et al. | Leiomyosarcoma  Liposarcoma | Retroperitoneum | 100 | 30 | 15 | 52 | 40% female | Median OS 87,7 months |
| Important prognostic factors in leiomyosarcoma survival: a National Cancer Database (NCDB) analysis. | 2020 | Gootee, J et al. | Leiomyosarcoma | ﻿Abdomen  Head and neck  Thorax and lung  Extremities  Female reproductive organs  Pelvis | 7154 | 7154 | 8 | 58 | 68% female | Median OS 95 months  5 year 60 %  10 year 45 % |
| Multidisciplinary Oncovascular Surgery is Safe and Effective in the Treatment of Intra-abdominal and Retroperitoneal Sarcomas: A Retrospective Single Centre Cohort Study and a Comprehensive Literature Review. | 2020 | Homsy, Pauliina et al. | Leiomyosarcoma  Liposarcoma  Other sarcomas | Abdomen  Retroperitoneum | 17 | 10 | NA | /59 | 76% female | Median OS 66 months  3 year 80% |
| Incidence and time trends of sarcoma (2000-2013): results from the French network of cancer registries (FRANCIM). | 2020 | Amadeo, Brice et al. | Leiomyosarcoma  Liposarcoma  GIST | Abdomen  Retroperitoneum  Female reproductive organs  Skin  Extremities | 3943 | 551 | NA | /63 | 63% female | NA |
| Recurrent Leiomyosarcoma of the Small Bowel: A Case Series. | 2020 | Ferrari, Cecilia et al. | Leiomyosarcoma | Abdomen  (Small bowel) | 4 | 4 | 11 | 79/74 | 50% female | Mean OS 33 months |
| Inoperable Primary Retroperitoneal Sarcomas: Clinical Characteristics and Reasons Against Resection at a Single Referral Institution | 2020 | Perhavec, Andraz et al. |  | Retroperitoneum | 322 | 60 | Operated patient 20  Non-operated 12,5 | /66 | 43% female | Operated patients 4 year OS 83,4%  Non-operated 10,3% |
| Polytetrafluoroethylene Is a Safe and Effective Interposition Conduit for Caval Reconstruction After Resection of Primary Leiomyosarcoma of the Inferior Vena Cava. | 2019 | Kalluri, Aravind G et al. | Leiomyosarcoma | Retroperitoneum  (Inferior vena cava) | 4 | 4 | 7,5 | 57/58 | 75% female | NA |
| Soft tissue leiomyosarcoma-diagnostics, management, and prognosis: Data of the registry cancer of the center of Tunisia. | 2019 | Mestiri, Sarra et al. | Leiomyosarcoma | ﻿Abdomen  Retroperitoneum  Extremities  Head and neck  Thorax | 29 | 29 | 9,4 | /52 | 48% female | 5 year OS 24% |
| Retroperitoneal Sarcomas: Does Laterality Matter? | 2019 | Johanna, Lou et al. | Leiomyosarcoma  Liposarcoma | Retroperitoneum | 106 | 28 | 91% bigger than 5 cm | 59 | 58% female | Median overall survival 47,5 months |
| Systematic review of anorectal leiomyosarcoma: Current challenges and recent advances. | 2019 | Nassif, MO et al. | Leiomyosarcoma | Abdomen | 51 | 51 | 6 | 60 | 51% female | 2 year survival 58 % |
| Incidence of soft tissue sarcoma in Taiwan: A nationwide population-based study (2007-2013). | 2019 | Hung, GY et al. | Leiomyosarcoma  Liposarcoma  GIST  Other sarcomas | ﻿Abdomen  Retroperitoneum  Connective tissues  Skin  Oral cavity and pharynx  Brain  Larynx, trachea, bronchus, and Thorax  Ear and nose  Breast  Kidney and urinary system  Male genital organs  Female reproductive organs  Lungs and pleura  Testis  Eye | 11393 | 605 | NA | NA | 59% female | NA |
| Liver Resection for Hepatic Metastases from Soft Tissue Sarcoma: A Nationwide Study. | 2019 | Grimme, FAB et al. | Leiomyosarcoma  Other | Liver (metastases)  Primary tumour:  Abdomen  Extra-abdominal | 38 | 24 | NA | 57 | 60% female | Median overall survival 46 months  1 year 88 %  3 year 54 %  5 year 41 % |
| Surgical Management of Primary Retroperitoneal Sarcomas: Rationale for Selective Organ Resection. | 2018 | Fairweather, Mark et al. | Leiomyosarcoma  Liposarcoma | Retroperitoneum | 118 | 27 | 15,5 | /59 | 50% female | NA |
| A contemporary analysis of radiotherapy effect in surgically treated retroperitoneal sarcoma. | 2018 | Nazzani, Sebastiano et al. | Leiomyosarcomas  Liposarcoma | ﻿Retroperitoneum | 1226 | 300 | 17 | /61 | 52% female | 5 year 62% |
| Retrospective Analysis of Outcome of Patients with Metastatic Leiomyosarcoma in a Tertiary Referral Center. | 2018 | van Cann, T et al. | Leiomyosarcoma | Abdomen  Extremities  Uterus | 122 | 35 | NA | /60 | 63% female | Median overall survival 16,1 months |
| Needle tract seeding following core biopsies in retroperitoneal sarcoma. | 2017 | van Houdt, WJ et al. | Leiomyosarcomas  Liposarcoma | Retroperitoneum | 498 | 89 | 23 | /60 | 47% female | Approximate 3 year survival 66% |
| Outcomes of Primary Colorectal Sarcoma: A National Cancer Data Base (NCDB) Review. | 2017 | Thiels, CA et al. | Leiomyosarcoma | Abdomen | 433 | 249 | 12% < 3  88% > 3 | /60 | 49% female | 5 year OS 44% |
| Intensity modulated radiation therapy and surgery for Management of Retroperitoneal Sarcomas: a single-institution experience. | 2017 | Cosper, PF et al. | Leiomyosarcomas  Liposarcoma | Retroperitoneum | 30 | 10 | 14 | /59 | 50% female | 3 year OS 68%  5 year OS 50% |
| Comparison between retroperitoneal leiomyosarcoma and dedifferentiated liposarcoma. | 2017 | Ishii, Takeaki et al. | Leiomyosarcomas  Liposarcoma | Retroperitoneum | 20 | 20 | 14 | /64 | 40% female | 2 year OS 69% |
| Gastrointestinal (GI) leiomyosarcoma (LMS) case series and review on diagnosis, management, and prognosis. | 2016 | Hilal, Lara et al. | Leiomyosarcoma | Abdomen | 11 | 11 | 9,7 | /56% | 45% female | 5 year OS 22% |
| Nonrhabdomyosarcomatous abdominopelvic sarcomas: Analysis of prognostic factors. | 2016 | Iqbal, Nida et al. | Leiomyosarcoma  Liposarcoma | Retroperitoneum | 88 | 32 | 12 | /40 | 58% female | 5 year OS 42%  Median OS 43 months |
| Multidetector computed tomography features of pancreatic metastases from leiomyosarcoma: Experience at a tertiary cancer center. | 2016 | Suh, CH et al. | Leiomyosarcoma | Pancreas (metastases)  Primary tumour: Retroperitoneum  Uterine  Extremities | 13 | 13 | 2 | 57 | 85% female | Median OS 13 months |
| Long-term outcomes in treatment of retroperitoneal sarcomas: A 15 year single-institution evaluation of prognostic features. | 2016 | Abdelfatah, Eihab et al. | Leiomyosarcoma  Liposarcoma | Retroperitoneum | 131 | 52 | 12 | /59 | 51% female | Median OS 48,7 months |
| Management of Tumors of the Ischiorectal Fossa: The Role of Percutaneous Biopsy. | 2015 | Buchs, NC et al. | Leiomyosarcoma  Liposarcoma | Abdomen | 11 | 2 | 5 | /50 | 72% female | NA |
| Leiomyosarcoma: One disease or distinct biologic entities based on site of origin? | 2015 | Worhunsky, DJ et al. | Leiomyosarcoma | Retroperitoneum  Uterus  Trunk  Extremities | 138 | 49 | 11 | /57 | 71% female | NA |
| Leiomyosarcoma: investigation of prognostic factors for risk-stratification model. | 2015 | Kim, Hyun Ju et al. | Leiomyosarcoma | Abdomen  Retroperitoneum  Pelvis  Extremities  Thorax  Head and neck | 129 | 42 | 10,5 | /56 | 67% female | 5 year OS 77%  10 year OS 63% |
| Leiomyosarcoma with alternative lengthening of telomeres is associated with aggressive histologic features, loss of ATRX expression, and poor clinical outcome. | 2015 | Liau, JY et al. | Leiomyosarcoma | Retroperitoneum  Abdomen  Uterus | 92 | 29 | 10,1 | 55/ | 85% female | 2 year OS 46% |
| Liver resection for metastatic colorectal leiomyosarcoma: a single center experience. | 2015 | Faraj, Walid et al. | Leiomyosarcoma | Liver (metasteses)  Primary tumour:  Abdomen | 5 | 5 | NA | /47 | 60% femlae | Median OS 47 months |
| Contemporary management and classification of hepatic leiomyosarcoma. | 2015 | Hamed, MO et al. | Leiomyosarcoma | Abdomen  Liver (both primary and metastases) | 8 | 8 | NA | /58 | 87% female | 1 year OS 100%  3 year OS 75%  5 year OS 50%  Median OS 56 months |
| Lessons learned from the study of 10,000 patients with soft tissue sarcoma. | 2014 | Brennan, Murray F et al. | Leiomyosarcoma  Liposarcoma  Fibrosarcoma  GIST  Synovial sarcoma  ﻿Malignant peripheral nerve sheath tumor  ﻿Undifferentiated pleomorphic sarcoma  Myxofibrosarcoma | Abdomen  Retroperitoneum  Viscera  Extremities  Trunk  Other | 10000 | Approximately 940 (number from graph) | < 5 32%  5-10 30%  >10 38% | NA | 51% female | Approximately  5 year DSS 50% (number from graph) |
| Predictors of survival and recurrence in primary leiomyosarcoma. | 2013 | Gladdy, Rebecca A et al. | Leiomyosarcoma | Abdomen  Retroperitoneum  Extremity  Truncal | 353 | 144 | 6 | /57 | 44% female | 5 year DSS 67%  50 months |
| Gemcitabine and docetaxel for metastatic soft tissue sarcoma - a single center experience. | 2013 | Schmitt, Thomas et al. | Leiomyosarcoma  Liposarcoma  Pleiomorphic sarcoma | Abdomen  Retroperitoneum  Extremities | 34 | 5 | NA | /59 | 41% female | Median OS 15,3 months |
| Primary small bowel malignancy: a 10-year clinical experience from Southern Taiwan. | 2013 | Lin, CY et al. | Leiomyosarcoma Other malignancies | Abdomen | 49 | 3 | NA | /58 | 31% female | 5 year survival 57,1% |
| Clinicopathological features of primary leiomyosarcoma of the gastrointestinal tract following recognition of gastrointestinal stromal tumours. | 2013 | Yamamoto, Hidetaka et al. | Leiomyosarcoma | Abdomen | 7 | 7 | 8,5 | 78 | 43% female | 5 year OS 51,6 months |
| Incidence of soft tissue sarcoma and beyond: a population-based prospective study in 3 European regions. | 2012 | Mastrangelo, Giuseppe et al. | Leiomyosarcoma | Abdomen  Retroperitoneum  Extremities  Head and neck  Trunk | 252 | 96 (including female reproductive organs) | NA | NA | 67% female | NA |
| Primary leiomyosarcomas of the gastrointestinal tract in the post-gastrointestinal stromal tumor era. | 2012 | Aggarwal, Gitika et al. | Leiomyosarcoma | Abdomen | 55 | 55 | 7,2 | /60 | 47% female | Median OS 33 months |
| Dedifferentiated leiomyosarcoma: clinicopathological analysis of 18 cases. | 2011 | Chen, Eleanor et al. | Leiomyosarcoma | Retroperitoneum  Extremities  Trunk  Uterus  Prostate | 18 | 8 | 12 | /61 | 63% female | NA |
| Liver resection for metastatic soft tissue sarcoma: an analysis of prognostic factors. | 2011 | Marudanayagam, R et al. | Leiomyosarcoma | Abdomen  Retroperitoneum  Thorax  Uterus  Extremities  Skin | 36 | 20 | NA | /58 | 64% female | 1 year OS 90,3%  3 year OS 48%  5 year OS 31,8% |
| Proton-beam, intensity-modulated, and/or intraoperative electron radiation therapy combined with aggressive anterior surgical resection for retroperitoneal sarcomas. | 2010 | Yoon, SS et al. | Leiomyosarcoma  Liposarcoma | Retroperitoneum | 28 | 6 | 9,75 | /56 | 54% female | 3 year OS 87% |
| Abdominal soft tissue sarcoma: a multicenter retrospective study. | 2010 | Nishimura, Junichi et al. | Leiomyosarcoma  Liposarcoma | Abdomen  Retroperitoneum | 82 | 32 | 8 | /54 | 72% female | 1 year OS 92,1%  3 year OS 68,6%  5 year OS 62,2% |
| Leiomyosarcoma of intravascular origin--a rare tumor entity: clinical pathological study of twelve cases. | 2010 | Tilkorn, DJ et al. | Leiomyosarcoma | Retroperitoneum  (Vena Cava)  Extremities | 182 | 12 | 7,4 | /59 | 75% female | 3 year OS 57% |
| Intraabdominal and retroperitoneal soft-tissue sarcomas--outcome of surgical treatment in primary and recurrent tumors. | 2010 | Sogaard, Ane S et al. | Leiomyosarcoma  Liposarcoma  GIST | Abdomen  Retroperitoneum | 87 | 13 |  |  | 51% female | Primary tumour 5 year OS 70,2%  Recurrent disease 5 year OS 51,8% |
| Transanal endoscopic microsurgery for rectal neoplasms: experience of 300 consecutive cases. | 2009 | Allaix, ME et al. | Leiomyosarcoma | Abdomen | 300 | 1 | NA | /63 | 38% female | 5 year OS 87,6% |
| Gallbladder sarcoma: a clinicopathological study of seven cases from the UK and Austria with emphasis on morphological subtypes. | 2009 | Husain, EA et al. | ﻿Leiomyosarcoma  Malignant fibrous histiocytoma  Storiform pleo- morphic sarcoma  Angiosarcoma  Liposarcomas | Abdomen | 7 | 1 | 2,5 | /70 | 86% female | NA |
| True smooth muscle tumors of the small intestine: a clinicopathologic, immunhistochemical, and molecular genetic study of 25 cases. | 2009 | Miettinen, Markku et al. | Leiomyosarcoma  Leiomyoma | Abdomen | 25 | 16 | 8 | /62 | 38% female | Median OS 41 months |
| Intraoperative radiation therapy for locally advanced and recurrent soft-tissue sarcomas in adults. | 2008 | Tran, PT et al. | ﻿Leiomyosarcoma  Liposarcoma  Epithelioid sarcoma  Fibrosarcoma  Nerve sheath sarcoma  Malignant fibrous histiocytoma Synovial Sarcoma | Retroperitoneum  Pelvis  Extremity  Other | 39 | 11 | NA | 53/ | 50% female | 5 year DSS 30% |
| Outcomes for soft-tissue sarcoma in 8249 cases from a large state cancer registry. | 2007 | Gutierrez, JC et al. | Leiomyosarcoma  GIST  Malignant fibrous histiocytoma Fibrosarcoma | Abdomen  Retroperitoneum  Extremities  Head or neck | 8249 | Approx. 3000  (viscera, retroperitoneum and trunk) | Retroperitoneum: 60% >10cm  Trunk: 35% >10 cm | /66 | 51% female | For leiomyosarcoma and GIST: 5 year OS 18,7%  10 year OS 6% |
| Surgical treatment of retroperitoneal leiomyosarcoma with adjuvant radiotherapy. | 2007 | Tufek, Iiter et al. | Leiomyosarcoma | Retroperitoneum | 2 | 2 | 1 tumour 3 cm  Other NA | 42/ | 50% female | 3 years OS 100% |
| Results of a single-center experience with resection and ablation for sarcoma metastatic to the liver. | 2006 | Pawlik, TM et al. | Leiomyosarcoma  GIST  Unclassified | Liver (metastases)  Primary tumour:  Abdomen  Retroperitoneum  Uterus | 66 | 18 | 3,9 | /54 | 50% female | Median OS 47 months  1 year OS 91,2%  3 year OS 65,4%  5 year OS 27,1% |
| Retroperitoneal soft tissue sarcomas: prognosis and treatment of primary and recurrent disease in 117 patients. | 2006 | Alldinger, Ingo et al. | Leiomyosarcoma  Liposarcoma  Malignant Schwannoma  MFH  Chondrosarcoma  Rhabdomyosarcoma Fibrosarcoma  Myxoid sarcoma  Pleomorph sarcoma  Neurofibrosarcoma Ganglioneuroblastoma  Synovial sarcoma | Retroperitoneum | 117 | 23 | Tumour >5cm in 98% | NA | 49,6% female | Median survival 45 months |
| Malignant smooth muscle tumours of soft tissue--a demographic and clinicopathological study at a tertiary care hospital. | 2005 | Shah, Hamidullah et al. | ﻿Leiomyosarcoma | Abdomen  Pelvis  Head and Neck  Extremities  Chest | 205 | 53 | Mean 7,23 | 48,4 | 50% female | NA |
| Small bowel tumours: a 10 year experience in four Sydney teaching hospitals. | 2004 | Rangiah, DS et al. | Leiomyosarcoma  Adenocarcinoma  Carcinoid tumour  Lymphoma  Benign causes | Abdomen  (Small bowel) | 166 | 9 | NA | /69 | 48% female | NA |
| Clinical analysis of primary small intestinal disease: A report of 309 cases. | 2004 | Zhan, Jun et al. | Leiomyosarcoma  Adenocarcinoma  Lymphoma  Benign causes | Abdomen  (Small bowel) | 309 | 34 | NA | 51,2 | 46% female | NA |
| Gastrointestinal stromal tumors, intramural leiomyomas, and leiomyosarcomas in the duodenum: a clinicopathologic, immunohistochemical, and molecular genetic study of 167 cases. | 2003 | Miettinen, Markku et al. | Leiomyosarcoma  GIST  Leiomyoma | Abdomen  (duodenum) | 167 | 5 | 13 | 54/55 | 40% female | 3 year survival 100%  5 year survival 80% |
| Gastrointestinal stromal tumors and leiomyosarcoma of the abdomen and retroperitoneum: a clinical comparison. | 2001 | Clary, BM et al. | Leiomyosarcoma  GIST | Retroperitoneum  Abdomen | 322 | 136 | <5 12%  5-10 38%  >10 26% | /54 | 69% female | Median OS complete resection 55 months.  Incomplete  12 months |
| Small bowel sarcoma: analysis of survival from the National Cancer Data Base. | 2001 | Howe, JR et al. | Leiomyosarcoma  ﻿Epithelioid sarcoma  Kaposi’s sarcoma Sarcoma  NOS  Spindle cell sarcoma  Fibrous histiocytoma  Malignant neurolemmoma  Other | Abdomen  (Small bowel) | 1441 | 1082 | <5 30%  5-10 41%  >10 29% | /59,3 | 46% female | 1 year DSS 81,8%  2 year DSS 63,0%  3 year DSS 51,0%  4 year DSS 44,7%  5 year DSS 40,3% |
| Sarcomas metastatic to the liver: response and survival after cisplatin, doxorubicin, mitomycin-C, Ethiodol, and polyvinyl alcohol chemoembolization. | 2001 | Rajan, DK et al. | Leiomyosarcoma  Angiosarcoma  Malignant fibrous histiocytoma | Liver (metastases)  Abdomen (primary tumours) | 11 | 2 | NA | NA | 50% female | 1 year CS 81%  2 year CS 54%  3 year CS 40%  Median survival 20 months |
| Gastrointestinal stromal tumors, intramural leiomyomas, and leiomyosarcomas in the rectum and anus: a clinicopathologic, immunohistochemical, and molecular genetic study of 144 cases. | 2001 | Miettinen, Markku et al. | Leiomyosarcoma  GIST  Leiomyoma | Abdomen  (Rectum and anus) | 196 | 8 | 3 | 54/58 | 75% female | 1 year survival 100%  3 year survival 86%  5 year survival 57% |
| Evaluation of putative molecular biomarkers in abdominal and retroperitoneal leiomyosarcomas. | 2001 | Shpitz, B et al. | Leiomyosarcoma | Abdomen  Retroperitoneum | 43 | 29 | <5 7%  5-10 40%  >10 33% | 62/ | 48% female | 5 year DFS 47% |
| Malignant tumors of the small intestine: a review of 144 cases. | 2000 | North, JH et al. | Leiomyosarcoma  Adenocarcinoma  Carcinoid  Lymphoma | Abdomen  (Small bowel) | 144 | 18 | NA | 57/ | 50% female | 5 year survival 22% |
| Esophageal stromal tumors: a clinicopathologic, immunohistochemical, and molecular genetic study of 17 cases and comparison with esophageal leiomyomas and leiomyosarcomas. | 2000 | Miettinen, Markku et al. | Leiomyosarcoma  GIST  Leiomyoma | Abdomen  (Esophagus) | 68 | 3 | 9-16 | 65/62 | 29% female | 1 year survival 335  3 year survival 0% |
| Tumors of the small intestine | 2000 | Blanchard, DK et al. | Leiomyosarcoma  Leiomyoma | Abdomen  (Small bowel) | 1689 | 1689 | <5 22%  5-9 44%  10-14 22%  15-19 7%  >20 5% | /55 | 43% female | 5 year survival 27,8% |
| Primary small bowel malignancies: single-center results of three decades. | 2000 | Ojha, A et al. | Leiomyosarcoma  Adenocarcinoma  Lymphoma  Neurogenic tumour  Unclassified carcinoma  Other sarcoma | Abdomen  (Small bowel) | 64 | 8 | NA | /57 | 50% female | Approx. (from graph)  1 year survival 67%  3 year 50%  5 year 30% |
| Surgical outcome after curative resection of rectal leiomyosarcoma. | 2000 | Yeh, CY et al. | Leiomyosarcoma | Abdomen  (Rectum) | 40 | 40 | 6,8 | 59/ | 48% female | Wide local excision 5 year DFS 32%  Radical resection 52% |
| Malignancy risk prediction for primary jejunum-ileal tumors. | 2000 | Marques, RG et al. | Leiomyosarcoma  Adenocarcinoma  Carcinoids  Lymphoma  Benign tumours | Abdomen  (Small bowel) | 27 | 7 | NA | 53/ | 59% female | NA |
| Gastrointestinal stromal tumors and leiomyosarcomas in the colon: a clinicopathologic, immunohistochemical, and molecular genetic study of 44 cases. | 2000 | Miettinen, Markku et al. | Leiomyosarcoma  GIST | Abdomen  (Colon) | 35 | 7 | 6,5 | 58/61 | 29% female | 1 year OS 71%  3 year OS 57%  5 year OS 29% |
| Tumors of the appendix and colon. | 2000 | Hatch, KF et al. | Leiomyosarcoma | Abdomen  (Colon and appendix) | 257 | 98 | <5 15%  5-9 51%  10-14 19%  >15 16% | Most patient 50-59 years | 48% female | NA |
| Radiological features of leiomyomatous tumors of the colon and rectum. | 2000 | Lee, SH et al. | Leiomyosarcoma | Abdomen  (Rectum) | 12 | 10 | 8,8 | 49/ | 33% female | NA |
| Soft tissue leiomyosarcomas and malignant gastrointestinal stromal tumors: differences in clinical outcome and expression of multidrug resistance proteins. | 2000 | Plaat, BE et al. | Leiomyosarcoma  GIST | Retroperitoneum  Extremities  Unspecified location | 29 | 4 | NA | /58 | 75% female | Mean OS 20 months  Median OS 17,5 months |
| Tumors of the rectum and anal canal. | 2000 | Hatch, KF et al. | Leiomyosarcoma | Abdomen  (Rectum and anus) | 480 | 199 | <5 37%  5-9 50%  10-14 11%  >15 2% | 73% were 40-69 years | 41% female | 5 year survival 37,5% |
| Sphincter preservation of leiomyosarcoma of the rectum and anus with local excision and brachytherapy. | 1999 | Grann, A et al. | Leiomyosarcoma | Abdomen  (Rectum and anus) | 8 | 8 | 4,2 | 62 | 50% female | 3 year OS 71%  Median survival 53 months |
| Leiomyosarcoma of the inferior vena cava: prognosis and comparison with leiomyosarcoma of other anatomic sites. | 1999 | Hines, OJ et al. | Leiomyosarcoma | Retroperitoneum  (Inferior vena cava)  Abdomen  Uterus | 14 | 14 | 8,8 | /56 | 79% female | 3 year OS 88%  5 year OS 53%  10 year OS 53% |
| Retroperitoneal leiomyosarcomas unassociated with the gastrointestinal tract: a clinicopathologic analysis of 17 cases. | 1999 | Rajani, B et al. | Leiomyosarcoma | Retroperitoneum  Abdomen | 17 | 17 | 13 | /60 | 94% female | 1 year OS 88%  3 year OS 65%  5 year OS 35% |
| Small bowel tumors: diagnosis, therapy and prognostic factors. | 1999 | Naef, M et al. | Leiomyosarcoma  Adenocarcinoma  Carcinoid  Benign lesions | Abdomen  (Small bowel) | 42 | 7 | NA | 61 | 44% female | 1 year OS 43%  5 year OS 21%  Median OS 26,9 months |
| Colorectal sarcoma: analysis of failure patterns. | 1998 | Luna-Pérez, P et al. | Leiomyosarcoma  Malignant fibrous histiocytoma | Abdomen  (Colon and rectum) | 13 | 9 | 8 | 54 | 39% female | 5 year OS 40% |
| Prognostic factors in resected primary small bowel tumors. | 1998 | Brücher, BL et al. | Leiomyosarcoma  Adenocarcinoma  Neuroendocrine tumour  Lymphoma  Unclassified  Benign tumours | Abdomen  (Small bowel) | 71 | 5 | NA | 59/ | 44% female | Median survival 31,8 months |
| Prosthetic replacement of the inferior vena cava for malignancy. | 1998 | Sarkar, R et al. | ﻿Leiomyosarcoma Teratoma  Renal cell carcinoma Lymphoma | Retroperitoneum  (Inferior vena cava) | 10 | 7 | NA | /56 | 86% female | Median survival 21 months |
| Complete hepatic resection of metastases from leiomyosarcoma prolongs survival. | 1998 | Chen, H et al. | Leiomyosarcoma | Liver (metastases)  Primary tumour of Abdomen  Retroperitoneum  Female reproductive organs | 11 | 11 | 3,8 | 56/57 | 82% female | Median survival 39 months |
| The effect of extend of caval resection in the treatment of inferior vena cava leiomyosarcoma. | 1997 | Mingoli, A et al. | Leiomyosarcoma | Retroperitoneum  (Inferior vena cava) | 120 | 120 | Mean 11 | 55/55 | 88% female | 5 year survival 49%  10 year survival 29% |
| Smooth muscle tumors of the gastrointestinal tract: analysis of prognostic factors. | 1996 | Chou, FF et al. | Leiomyosarcoma  Leiomyoma | Abdomen | 80 | 45 | Mean 11 | 57/ | 49% female | 1year OS 79,7%  3 year OS 48,6%  5 year OS 19,6%  Median survival 34 months |
| Retroperitoneal leiomyosarcoma: eight cases and a literature review. | 1995 | Todd, C S et al. | Leiomyosarcoma | Retroperitoneum  Uterus | 8 | 8 | 14,6 | 61/ | 100% female | Median OS 18 months |
| Malignant soft-tissue tumors in a large referral population: distribution of diagnoses by age, sex, and location. | 1995 | Kransdorf, MJ | Leiomyosarcoma  Malignant fibrous histiocytoma  Liposarcoma  Malignant schwannoma  Dermatofibrosarcoma  Synovial sarcoma  Fibrosarcoma | Retroperitoneum  Extremities  Trunk  Head and neck | 1039 | 338 | NA | 58/ | 51% female | NA |
| Small bowel cancer: a 30-year review. | 1994 | Frost, DB et al. | Leiomyosarcoma  Adenocarcinoma  Lymphoma  Carcinoid tumours | Abdomen  (Small bowel) | 69 | 7 | NA | /63 | 49% female | 10 year survival 20% |
| Resectable retroperitoneal soft tissue sarcomas. The effect of extent of resection and postoperative radiation therapy on local tumor control. | 1994 | van Doorn, RC et al. | Leiomyosarcoma  Liposarcoma  Malignant fibrous histiocytoma  Malignant Schwannoma  Fibrosarcoma  Rhabdomyosarcoma | Retroperitoneum | 34 | 10 | NA | 57/ | 59% female | 5 year survival 35% |
| Small bowel tumors: an analysis of tumor-like lesions, benign and malignant neoplasms. | 1994 | Matsuo, S et al. | Leiomyosarcoma  Adenocarcinoma  Lymphoma  Benign tumours | Abdomen  (Small bowel) | 33 | 9 | 12,2 | 62/ | 27% female | Mean survival 17,2 months |
| Primary small bowel malignant tumors. | 1994 | Garcia Marcilla, JA et al. | Leiomyosarcoma  Lymphoma  Adenocarcinoma  Carcinoid tumour | Abdomen  (Small bowel) | 69 | 7 | NA | 53/ | 33% female | 5 year OS 43% |
| Leiomyosarcomas of the gastrointestinal tract. | 1994 | Hansen, CP | Leiomyosarcoma | Abdomen | 13 | 13 | 5 | /69 | 31% female | NA |
| Colonic submucosal tumors: a new classification based on radiologic characteristics. | 1993 | Kawamoto, K et al. | Leiomyosarcoma  Carcinoids  Lymphoma  Lipoma  Lymphangioma Leiomyoma  Unspecified lesions | Abdomen  (Colon) | 89 | 6 | NA | 56/ | 48% female | NA |
| Management of primary sarcomas of the retroperitoneum. | 1993 | Rossi, CR et al | Leiomyosarcoma  Liposarcoma  Unspecified lesions | Retroperitoneum | 25 | 10 | Mean 15 cm | 49/ | 68% female | 10 year survival 33% |
| Leiomyosarcoma of the rectum and anal canal. | 1993 | Tjandra, J J et al. | Leiomyosarcoma | Abdomen  (Anus and rectum) | 8 | 8 | 4 | /63 | 63% female | Media survival 24 months |
| Smooth muscle tumours of the stomach: clinicopathological aspects. | 1993 | Halpin, R et al. | Leiomyosarcoma  Leiomyoma  Leiomyoblastoma  Other | Abdomen  (Stomach) | 21 | 8 | NA | 62/ | 63% | NA |
| Primary neoplasms of the small bowel. | 1992 | Serour, F et al. | Leiomyosarcoma  Lymphoma  Adenocarcinoma | Abdomen  (Small bowel) | 44 | 10 | NA | 53/ | 52% female | 1 year OS 78%  2 year OS 78% |
| Smooth muscle tumors of the gastrointestinal tract. A 10-year experience. | 1992 | Spiliotis, J et al. | Leiomyosarcoma  Leiomyoma | Abdomen | 32 | 5 | 9,9 | 62/ | 50% female | 3 year survival rate 50%  5 year survival rate 0% |
| Primary malignant tumors of the small intestine: analysis of 40 Japanese patients. | 1992 | Kusumoto, H et al. | Leiomyosarcoma  Adenocarcinoma  Lymphoma  Carcinoid tumour | Abdomen  (Small bowel) | 40 | 8 | NA | 65/ | 50% female | 5 year survival rate 57% |
| Dedifferentiated leiomyosarcoma of the intestinal tract: histological, ultrastructural and immunohistochemical examinations. | 1992 | Fukuda, T et al. | Leiomyosarcoma | Abdomen  (Small bowel and colon) | 6 | 6 | 4 | 65/65 | 33% female | NA |
| Colorectal leiomyosarcomas: a pathobiologic study with long-term follow-up. | 1992 | Friesen, R et al. | Leiomyosarcoma | Abdomen  (Colon and rectum) | 12 | 12 | NA | 65/ | 8% female | 1 year survival 91,6%  3 year survival 83%  5 year survival 67% |
| Surgery for disseminated abdominal sarcoma. | 1992 | Karakousis, CP et al. | Leiomyosarcoma  Liposarcoma  Malignant fibrous histiocytoma  Malignant mesenchymoma  Other sarcomas | Abdomen  Retroperitoneum  Abdominal wall | 72 | 45 | 14 | 51/55 | 54% female | Mean survival 39 months |
| Sarcomas in north west England: III. Survival. | 1992 | Hartley, AL et al. | Leiomyosarcoma  Malignant fibrous histiocytoma Liposarcoma  Malignant schwannoma  Rhabdomyosarcoma  Synovial sarcoma  Fibrosarcoma  Angiosarcoma  Chondrosarcoma  Epithelioid sarcoma  Clear cell sarcoma | Abdomen  Retroperitoneum | 310 | 14  27 |  | 69  58 |  | 5 year relative survival  19  17 |
| Primary malignant tumor of the small intestine. | 1991 | Lee, WJ et al. | Leiomyosarcoma  Lymphoma  Adenocarcinoma  Carcinogen tumour | Abdomen  (Small bowel) | 100 | 26 | 10,4 | 51 | 46% female | 1 year survival rate 52%  5 year survival rate 33% |
| Soft tissue sarcomas in Iceland 1955-1988. Analysis of survival and prognostic factors. | 1991 | Baldursson, G et al. | ﻿Leiomyosarcoma  Malignant fibrous histiocytoma Liposarcoma  Malignant schwannoma  Rhabdomyosarcoma  Synovial sarcoma  Fibrosarcoma  Angiosarcoma  Chondrosarcoma  Epithelioid sarcoma  Clear cell sarcoma  Unclassified | Retroperitoneum  Abdomen  Extremities  Head and neck  Trunk | 21 | 21 (might include leiomyosarcomas outside the abdomen and retroperitoneum) | NA | 55 | 62% female | 5 year OS 38%  10 year OS 29% |
| Retroperitoneal sarcomas: pre-operative assessment and surgical therapy. | 1991 | Blanken, R et al. | Leiomyosarcoma  Liposarcoma  Other sarcomas | Retroperitoneum | 22 | 8 | All tumours >10 | 47/ | 41% female | OS 27,9 months |
| Smooth muscle tumours of the alimentary tract. | 1990 | Diamond, T et al. | Leiomyosarcoma  Leiomyoma | Abdomen | 51 | 7 | NA | 64/ | 29% female | 1 year survival 57%  2 year survival 43% |
| Abdominal leiomyosarcoma: aggressive management. | 1989 | Bowers, B A et al. | Leiomyosarcoma | Abdomen | 34 | 34 | NA | 55/ | 50% female | Median survival 29 months  5 year survival 16% |
| Leiomyosarcoma of the rectum and anus. A series of 22 cases. | 1989 | Randleman Jr, C D et al. | Leiomyosarcoma | Abdomen  (Rectum and anus) | 22 | 22 | NA | 59,5/ | 41% female | 1 year OS 90%  5 year OS 75%  10 year OS 51% |
| Sarcomas of the retroperitoneum and genitourinary tract. | 1989 | Zhang, G et al. | Leiomyosarcoma  Liposarcoma  Malignant fibrous histiocytoma  Other sarcomas | Retroperitoneum  Genitourinary tract | 62 | 13 | NA | 57/ | 39% female | 5 year survival 8% |
| Soft-tissue sarcoma: initial characteristics and prognostic factors in patients with and without metastatic disease. | 1988 | Torosian, MH et al. | Leiomyosarcoma  Liposarcoma  Malignant fibrous histiocytoma  Fibrosarcoma  Tenosynovial sarcoma  Other sarcomas | Retroperitoneum  Abdomen  Extremities  Head and neck  Breast | 124 | 113 (may include non-abdominal or retroperitoneal tumours) | <5 cm 27,5%  >5cm 72,5% | /49,4 | 46% female | 1 year survival in patients with metastases 39% |
| Soft tissue sarcomas in Osaka, Japan (1962-1985): review of 290 cases. | 1988 | Tsujimoto, M et al. | Leiomyosarcoma  Malignant fibrous histiocytoma Liposarcoma  Synovial sarcoma Rhabdomyosarcoma  Neurogenic sarcoma Fibrosarcoma  Other sarcomas | Retroperitoneum  Extremities  Trunk  Head and neck | 16 | 8 (may include non-abdominal or retroperitoneal tumours) | NA | 51/ | 45% female | NA |
| A clinicopathologic and immunohistochemical study of 16 patients with small intestinal leiomyosarcoma. Limited utility of immunophenotyping. | 1987 | Ricci Jr, A et al. | Leiomyosarcoma | Abdomen  (Small bowel) | 16 | 16 | Mean 8,2 | 61/ | 31% female | Grade I: 2 year survival 100%, 5 year survival 50%  Grade II: 2 year survival 80%, 5 year survival 33%  Grade III: 2 year survival 20%  5 year survival 0% |
| Smooth muscle tumours of the upper gastrointestinal tract. | 1987 | Senewiratne, S et al. | Leiomyosarcoma  Leiomyoblastoma  Leiomyoma | Abdomen | 46 | 10 | Mean 8 | 55/ | 58% | 1 year survival 60%  2 year survival |
| A new pattern of histologic predominance and distribution of malignant diseases of the small intestine. | 1987 | Taggart, DP et al. | Leiomyosarcoma  Lymphoma  Carcinoma  Carcinoid tumour | Abdomen | 106 | 5 | NA | NA | 80% female | NA |
| Leiomyosarcoma of the colon and rectum. | 1986 | Keane, PF et al. | Leiomyosarcoma | Abdomen  (Colon and rectum) | 3 | 3 | 1 tumour 5 cm | 72/73 | 67% female | 1 year survival 100% |
| Malignant smooth muscle tumors of the retroperitoneum and mesentery: a clinicopathologic analysis of 44 cases. | 1985 | Hashimoto, H et al. | Leiomyosarcoma  Epithelioid leiomyosarcoma | Retroperitoneum  Abdomen | 130 | 44 | <10 cm 11%  >10 cm 89% | /59 | 61% female | 5 year survival rate 21% |
| Smooth muscle tumors of the gastrointestinal tract. A study of 56 cases followed for a minimum of 10 years. | 1985 | Evans, HL | Leiomyosarcoma  Leiomyoma | Abdomen | 56 | 54 | <5 cm 12%  >5 cm 88% | NA | 44% female | Median survival high grade 25 months  Low grade 98 months |
| Primary retroperitoneal sarcomas. A review of 36 cases. | 1985 | Wist, E et al. | Leiomyosarcoma  Liposarcoma  Other sarcomas | Retroperitoneum | 36 | 15 | NA | 61/ | 53% female | Median survival 25 months |
| Combined modality management of local and disseminated adult soft tissue sarcomas: a review of 257 cases seen over 10 years at the Christie Hospital & Holt Radium Institute, Manchester. | 1985 | Bramwell, VH et al. | Leiomyosarcoma  Liposarcoma  Malignant fibrous histiocytoma  Fibrosarcoma  Neurfibrosarcoma  Other sarcomas | Abdomen  Retroperitoneum  Trunk  Uterine  Extremities | 257 | 46 | NA | /54 | 40% female | Viscera: 1 year survival 65%  3 year 10%  5 year 10%  Retroperitoneal:  1 year 60%  3 year 30%  5 year 10%  (Approximation from graph) |
| Primary small bowel malignancies. | 1985 | Johnson, AM et al. | Leiomyosarcoma  Adenocarcinoma  Carcinoid tumours | Abdomen  (Small bowel) | 55 | 12 | NA | 53/ | 33% female | 1 year survival 25%  5 year 25%  10 year 15% |
| Malignant tumors of the small bowel. | 1984 | Laws, HL et al. | Leiomyosarcoma  Adenocarcinoma  Lymphoma  Carcinoid  Fibrosarcoma | Abdomen  (Small bowel) | 45 | 9 | NA | NA | NA | NA |
| Myosarcomas of the small and large intestine: a clinicopathologic study. | 1983 | Shiu, MH et al. | Leiomyosarcoma  Malignant leiomyoblastoma | Abdomen | 38 | 34 | NA | Male /45  Female /59 | 39% female | 5 year survival 86% |
| Malignant tumors of the small intestine. | 1983 | Barclay, TH et al. | Leiomyosarcoma  Carcinoid  Adenocarcinoma | Abdomen | 209 | 19 | NA | NA | NA | 5 year OS 62% |
| Leiomyosarcoma of the gastro-intestinal tract: general pattern of metastasis and recurrence. | 1983 | Lee, YT | Leiomyosarcoma  Leiomyoma | Abdomen  Retroperitoneum | 3525 | 1214 | NA | 47/ | NA | Approximate 5 year OS 50% |
| Prognostic factors of 28 leiomyosarcomas of the small intestine. | 1982 | Chiotasso, PJ et al. | Leiomyosarcoma | Abdomen  (Small bowel) | 176 | 28 | Mean 7,7 | 53/ | 39% female | 5 year survival 29%  10 year survival 21% |
| Leiomyosarcoma of the rectum: the military experience. | 1981 | Feldtman, RW et al. | Leiomyosarcoma | Abdomen  (Rectum) | 7 | 7 | NA | 41/41 | 14% female | 1 year survival 86%  3 year survival 66%  5 year survival |
| A clinical review of small bowel neoplasms. | 1981 | Waterhouse, G et al. | Leiomyosarcoma  Carcinoid  Adenocarcinoma  Lymphoma | Abdomen  (Small bowel) | 70 | 9 | NA | NA | NA | Approximate 5 year survival 50% |
| Leiomyoma and leiomyosarcoma of the digestive tract--a report of 45 cases and review of the literature. | 1981 | Bruneton, JN et al. | Leiomyosarcoma  Leiomyoma | Abdomen | 45 | 9 | <5 cm 25%  5-10cm 50%  >5 cm 25% | 52/ | 22% female | NA |
| Leiomyosarcoma of soft tissue: a clinicopathologic study. | 1981 | Wile, AG et al. | Leiomyosarcoma | Retroperitoneum  Abdomen  Extremities  Trunk | 28 | 19 | <5 cm 11%  >5 cm 89% | 32%  50-59 years | 79% female | 5 year survival rate 35%  Median 43 months |
| A twenty-five year experience with primary malignant tumors of the small intestine. | 1980 | Awrich, AE et al. | Leiomyosarcoma  Carcinoid  Adenocarcinoma  Lymphoma  Fibrosarcoma | Abdomen  (Small bowel) | 82 | 9 | NA | 65/ | 35% female | 5 year survival 37%  Mean survival 13 months |
| Leiomyosarcoma of the colon and rectum. | 1980 | Stavorovsky, M et al. | Leiomyosarcoma | Abdomen  (Colon) | 5 | 5 | 12,5 | 54/54 | 80% female | NA |
| Leiomyosarcomas of the small intestine. | 1979 | Deck, KB et al. | Leiomyosarcoma | Abdomen  (Small bowel) | 20 | 20 | NA | 61 | 60% female | 5 year survival 42% |
| The small bowel tumor problem: an assessment based on a 20 year experience with 116 cases. | 1979 | Miles, RM et al. | Leiomyosarcoma  Carcinoid  Undifferentiated carcinoma  Lymphoma  Adenocarcinoma | Abdomen  (Small bowel) | 79 | 10 | NA | 30% 60-69 years | 47% female | 5 year survival 29%  10 year survival 17% |
| Smooth muscle tumors of the stomach and small bowel. | 1979 | Langley, JR et al. | Leiomyosarcoma  Leiomyoblastoma  Leiomyoma | Abdomen | 33 | 4 | <5 cm 50%  >5 cm 50% | 51-70 years | 100% female | NA |
| Leiomyosarcoma of the colon. | 1978 | Watson, LC et al. | Leiomyosarcoma | Abdomen  (Colon) | 37 | 37 | NA | 65% >40 years | 52% female | NA |
| Angiography of abdominal leiomyosarcoma. | 1978 | Granmayeh, M et al. | Leiomyosarcoma | Abdomen  Retroperitoneum  Genitourinary tract | 33 | 11 | NA | NA | 52% female | NA |
| Leiomyosarcoma of the small and large bowel. | 1978 | Akwari, OE et al. | Leiomyosarcoma | Abdomen | 108 | 108 | NA | 61% 40-60 years | 28% female | Curative resection  5 year 50%  10 year 35%.  Average survival palliative resection 27,4 months |
| Malignant tumors of the intestine: a review of 37 cases. | 1977 | Rich, JD | Leiomyosarcoma  Carcinoid tumour  Adenocarcinoma Lymphosarcoma  Reticulum cell sarcoma  Liposarcoma  Mesenchymal cell sarcoma | Abdomen  (Small bowel) | 37 | 5 | NA | 50 | 22% female | OS 25% |
| Leiomyosarcoma of the colon exclusive of the rectum. | 1975 | Tang, CK et al. | Leiomyosarcoma | Abdomen  (Colon) | 5 | 5 | Mean 14,7 | 60/57 | 60% female | 15 months survival 0% |
| Primary tumors of the small bowel. | 1975 | Treadwell, TA et al. | Leiomyosarcoma  Adenocarcinoma  Lymphosarcoma  Carcinoid tumour | Abdomen  (Small bowel) | 133 | 10 | NA | 55,9/ | 43% female | 5 year survival 30%  10 year survival 20% |
| Primary malignancies of the small bowel: a report of 96 cases and review of the literature. | 1974 | Wilson, JM et al. | Leiomyosarcoma  Adenocarcinoma  Carcinoid tumour | Abdomen  (Small bowel) | 96 | 11 | NA | 53/ | 47% female | 5 year survival 20% |

CS: cumulative survival, OS: overall survival, DSS: disease specific survival, DFS: disease free survival.
